# Acceptance of patients towards task‐autonomous robotic cochlear implantation: An exploratory study

**DOI:** 10.1002/rcs.2172

**Published:** 2020-10-04

**Authors:** Bernhard J. Jank, Markus Haas, Dominik Riss, Wolf‐Dieter Baumgartner

**Affiliations:** ^1^ Department of Otorhinolaryngology, Head and Neck Surgery Medical University of Vienna Vienna Austria

**Keywords:** cochlear implantation, image‐guided, patient acceptance, task‐autonomous robotic surgery

## Abstract

**Background:**

Recently, task‐autonomous image‐guided robotic cochlear implantation has been successfully completed in patients. However, no data exist on patients' perspective of this new technology. The aim of this study was to evaluate the acceptance of patients towards task‐autonomous robotic cochlear implantation (TARCI).

**Methods:**

We prospectively surveyed 63 subjects (51 patients and 12 parents of infants) scheduled for manual cochlear implantation. We collected sociodemographic and clinico‐pathological characteristics and their attitude towards TARCI for themselves or their child using a questionnaire. Differences between variables were analysed using one‐way analysis of variance and Spearman's rho was used to test for correlation.

**Results:**

Seventy‐three percent of patients and 84% of parents expressed a high acceptance towards TARCI for themselves, or their child, respectively. Interestingly, patients with a negative attitude towards TARCI were significantly younger.

**Conclusion:**

The attitude of patients and parents likely does not represent a barrier towards application of this new technology.

## 1 INTRODUCTION

Disabling hearing loss (HL) affects 466 million people worldwide, including 34 million children. Global cost of untreated HL is estimated at US $750 billion arising in part from loss of productivity due to higher unemployment rates in deaf people, necessary educational support for hearing impaired children and health care costs.^1^ While hearing aids are able to treat moderate HL, they remain ineffective in cases of severe impairments or deafness. In patients with damaged inner ear cells, but intact cochlear nerves, cochlear implants represent an effective treatment option. Since their approval by the FDA in the 1980s, more than 600,000 patients have undergone cochlear implants globally.^2^

Cochlear implantation is a microsurgical procedure that requires surgeons to operate in a sub‐millimetre range. The necessity to perform on the limits of human dexterity and tactile feedback poses a challenge to the avoidance of pressure transients and disturbances to the inner ear in order to preserve residual hearing.^3^ Currently, access to the inner ear for electrode implantation is achieved by performing mastoidectomy and posterior tympanotomy. In the pursuit of a less invasive approach, an image‐guided minimally invasive keyhole approach has been proposed, which eliminates the need for a mastoidectomy by drilling directly through the facial recess for inner ear access.^4^ By eliminating mastoidectomy, surgery duration and drilling time could potentially be reduced and the mastoid can be largely preserved.^5^

As the facial recess is bordered by important anatomical structures such as the facial nerve, the chorda tympani and the incus, an image‐guided approach is necessary to plan the drill trajectory based on individual patient anatomy in order to spare any damage to these structures. Noteworthy, the diameter of the facial recess is only 2.5 mm and needs to accommodate an electrode of ∼2 mm.^6^ This leaves 0.5 mm between drill and surrounding anatomy, requiring an absolute drilling accuracy of about 0.2 mm.^7^ To achieve this level of precision between planned and actual trajectory, task‐autonomous robotic platforms have been developed and were recently demonstrated to be feasible and safe.^7^, ^8^ Although no robotic platform to date allows for full cochlear implantation (i.e., task‐autonomous robotic drilling and electrode insertion combined), robotic systems for both tasks separately have been developed and have shown superiority compared to manual performance.^9^, ^10^, ^11^ If those tasks could be performed by one robotic platform, the possible benefits, such as optimized placement of the electrode and reduced invasiveness, would be considerable. It can be reasonably assumed that this technology will be available and surpass manual cochlear implantation in the near future.^12^

In contrast to robotic‐assisted surgery, which is already widely available and accepted for some procedures, such as prostatectomies^13^ and transoral surgery for head and neck diseases,^14^ task‐autonomous robotic platforms are merely monitored by the surgeon, and therefore represent a new category of surgical instrument. Evaluation of the level of acceptance of patients towards this new technology is therefore urgently needed before the technology becomes commercially available.

## 2 MATERIALS AND METHODS

### 2.1 Subjects

We prospectively recruited subjects among patients scheduled for manual cochlear implantation from April 2019 to March 2020 at the Department of Otorhinolaryngology and Head and Neck Surgery of the Medical University of Vienna, Austria. Exclusion criteria were insufficient German language skills. Patients older than 18 years were surveyed themselves (hereinafter referred to as “patients”), while for infants, their parents were surveyed (hereinafter referred to as “parents”). This study was approved by the Institutional Ethics Committee (EK No: 1176/2019).

### 2.2 Questionnaires

The questionnaire included a 12‐item section on sociodemographic data, followed by an 11‐item section on the acceptance of autonomous robotic cochlear implantation (Table 1). Possible answers were based on a four‐level Likert scale from “unlikely” to “likely.” The questionnaire and information sheet were distributed in person by a member of the study team (Bernhard J. Jank or Markus Haas), on the day prior to the implant surgery. The persons distributing the questionnaire and information sheet were not the surgeons performing the scheduled manual cochlear implantation to avoid bias. Subjects were invited to participate and in case of consent to the study, the background of task‐autonomous robotic cochlear implantation was explained based on a prepared information sheet along with informed consent to participate in the study. Responses were anonymized for further analysis.

**TABLE 1:** Items from the questionnaire

Q1	How likely would you have a robot drill the access to the inner ear?
Q2	Would you expect complications to be reduced if a robot is used?
Q3	Would you expect a shorter duration of surgery if a robot is used?
Q4	If you have anomalous anatomical conditions (e.g., deviating course of a nerve), would you tend to have surgery by the robot?
Q5	Do you feel well informed about the upcoming operation?
Q6	Would you accept an additional small intervention under local anaesthesia before the operation with a robot (implantation of the navigation screws)?
Q7	Do you think family members would advise against robotic surgery?
Q8	Would your relatives' opinion influence your decision?
Q9	If your surgeon advised you to perform the implantation using a robot, would you rather agree?
Q10	Would you discourage family members who need a cochlear implant from using a robot?
Q11	Is it important to you that the surgeon who monitors the robot can perform the cochlear implantation without a robot?

### 2.3 Audiometry

Pure‐tone audiometry was performed for adult patients and brainstem auditory evoked response was performed for infants scheduled for manual cochlear implantation. Air‐conduction thresholds were measured at 250–8000 Hz and bone conduction thresholds at 250–4000 Hz. A 110‐dB threshold was assumed to be the maximum level of HL. Pure tone average (PTA) was calculated as the mean hearing threshold at 500, 1000, 2000 and 3000 Hz. Residual hearing was defined as a hearing threshold below 80 dB in at least two neighbouring frequencies.^15^

### 2.4 Statistical analysis

Continuous variables were summarized as medians (25th–75th percentile) and categorical variables were summarized as absolute counts and percentages (%). Differences between categorical variables were analysed using two‐way tables and measures of association were performed using Pearson's chi‐squared or Fisher's exact test. Differences between continuous and categorical variables were analysed using one‐way analysis of variance (ANOVA). Spearman's rho was used to test for correlation. A two‐sided *p*‐value of <0.05 was considered as statistically significant. Statistical analysis was performed using STAT/IC 16.1 for Mac (Stata Corp) and Prism 8 for Mac was used to visualize data (GraphPad Software, LLC).

## 3 RESULTS

### 3.1 Demographic and clinico‐pathological characteristics

A total of 64 subjects met the inclusion criteria during the observation period. One subject withdrew consent and 63 subjects participated in the study. Eighty‐one percent (*n* = 51) of those subjects were patients receiving a cochlear implant themselves and 19% (*n* = 12) were parents of patients between the age of 0–10 years receiving a cochlear implant. Of all subjects, 54 % were female and it was the first ear surgery for 60%. Of the 25 patients who previously had ear surgery, 14 already had undergone cochlear implantation in the past. Thirty‐three subjects (52%) knew the aetiology of their HL, of which post‐infectious and idiopathic acute sensorineural HL were the most prevalent with 36 and 33%, respectively. Residual hearing was present in 20 patients (32%). Computed tomography of the petrous bone revealed pathologies or anomalous anatomy in 12 patients (19.6%) and magnetic resonance imaging revealed pathologies in two patients (3.7%). To analyse differences between parents and patients among study subjects, we performed two‐way tables with measures of association. Surveyed parents were significantly younger than patients (<30 years, 42% vs. 12%, *p* = 0.001), were less likely to live in a rural area (8% vs. 45%, *p* = 0.005) and had higher formal education (university degree, 42% vs. 10%, *p* = 0.001).

### 3.2 Acceptance towards autonomous robotic cochlear implantation

To evaluate the acceptance of subjects towards TARCI, we applied an 11‐item questionnaire based on the Unified Theory of Acceptance and Use of Technology (UTAUT; Table 1),^16^ asking for the level of acceptance, expectations and factors that would influence their decision.

**FIGURE 1:**
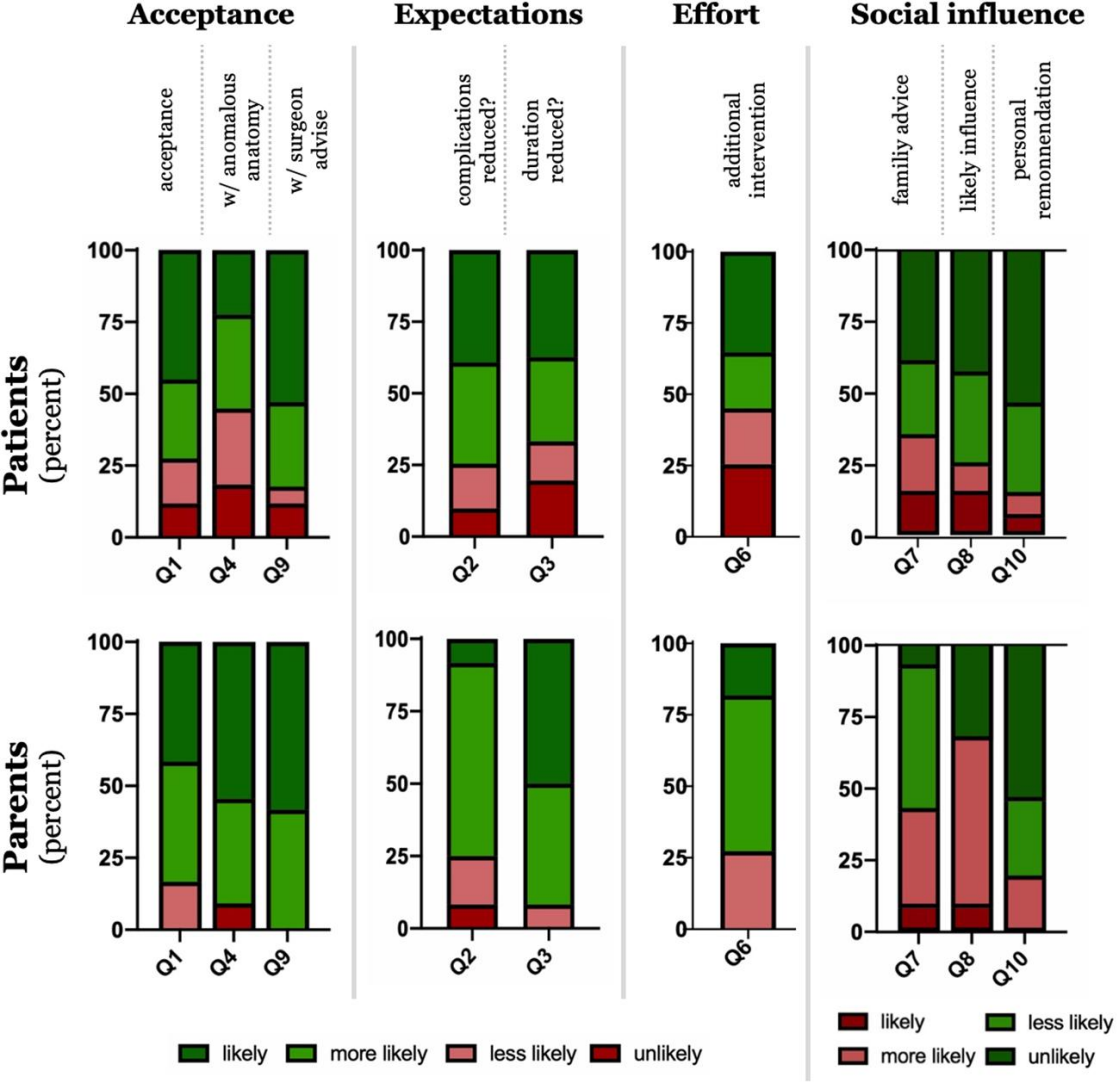
Acceptance of task‐autonomous robotic cochlear implantation. Levels of acceptance are displayed as percentages and separated for patients and parents. Categories of answers are defined based on the unified theory of acceptance and use of technology. Patients *n* = 51, parents *n* = 12

In general, 73% (37/51) of patients and 84% of parents (10/12) considered undergoing TARCI as more likely or likely. While six patients (12%) stated they would unlikely undergo TARCI, none of the parents excluded this option for their child (Figure 1‐Q1). Interestingly, in the hypothetical case of anomalous anatomy (i.e., deviant course of a nerve) the acceptance towards TARCI decreased in patients, while it became more likely for parents to choose TARCI for their child (Figure 1‐Q4). Next, we asked patients if they would be willing to undergo TARCI if the surgeon recommended this procedure for their case. Of those patients who would less likely or unlikely accept TARCI, 43% (6/14) would reconsider if their surgeon recommended it. Of the parents who would less likely accept TARCI (2/12), 100% would be willing to accept TARCI for their child if recommended (Figure 1‐Q9).

When asked for expectations of TARCI compared to conventional cochlear implantation patients expected a reduced complication rate (∼75% of patients and parents reported “likely” or “more likely”) and a shorter duration of surgery (66% of patients and 91% of parents; Figure 1‐Q2, Q3). As TARCI platforms navigate based on fiduciary markers, which have to be placed prior to image guided trajectory planning, we asked patients if they would be willing to undergo fiducial screw implantation under local anaesthesia prior to the implant surgery. Among patients who would agree to TARCI, approximately one‐third would not want to have fiducial screw implantation under local anaesthesia (Figure 1‐Q6). To analyse social influence on acceptance, we asked if subjects thought that family members would advise against TARCI. Thirty‐four percent of patients and 41 % of parents stated this as more likely or likely (Figure 1‐Q7). When asked if this advice would influence their decision, 24% of patients and 66% of parents answered with more likely or likely (Figure 1‐Q8). When we asked if they would advise against TARCI in case another family member needed that surgery, 13% of patients and 18% of parents answered with more likely or likely (Figure 1‐Q10).

Finally, we asked how important it would be that the surgeon who monitors the TARCI‐platform could perform the cochlear implantation via mastoidectomy himself. All subject stated this as very important (∼90% in both groups) or important (∼10%).

**FIGURE 2:**
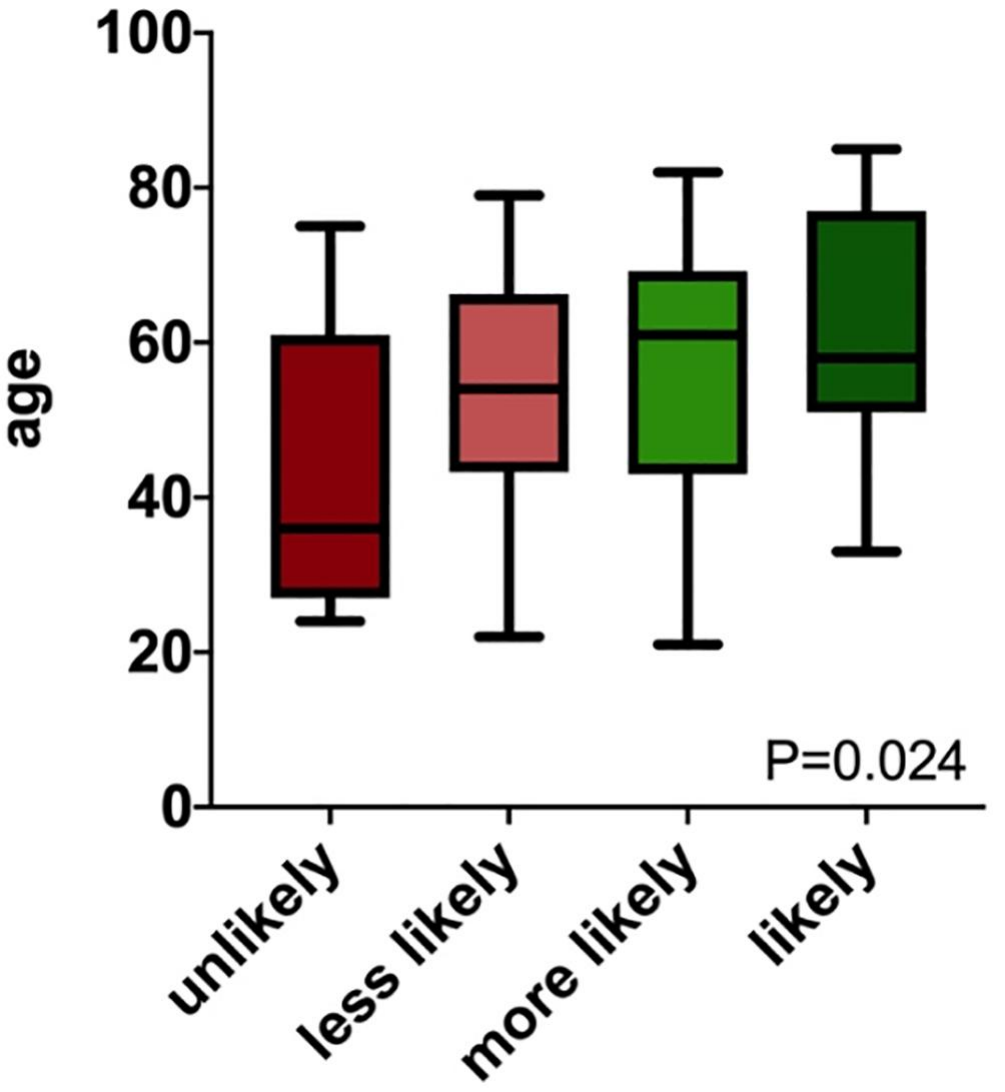
Box plot for patient age and level of acceptance. Patients with a negative attitude towards task‐autonomous robotic cochlear implantation (Q1) were significantly younger

In order to evaluate the influence of sociodemographic and clinico‐pathologic characteristics on the acceptance towards TARCI, we performed ANOVA for continuous variables and two‐way tables with measures of association for categorical variables. In patients, we found an association between age and acceptance towards TARCI. Interestingly, patients who were less willing to undergo TARCI were significantly younger than those who would likely take this option (mean ± SD in years; 35 ± 24.9 vs. 61 ± 15.2, *p* = 0.024, Spearman's *ρ* = 0.31, *p* = 0.029, Figure 2). No associations could be found for sex, area of residence, education, income, prior ear surgery, prior cochlear implantation or residual hearing with acceptance of TARCI.

## 4 DISCUSSION

This is the first study to investigate the acceptance of TARCI among patients and parents of infants undergoing cochlear implantation. We found the highest level of acceptance in parents, with no subject excluding the option of TARCI for their child. In patients, the level of acceptance was also high. However, about 16% of patients reported being unlikely to undergo TARCI, even if their surgeon would recommend it for their case.

Since the first application of a robotic system to assist in brain tumour biopsy in 1985,^17^ huge advancements have been made in robotics. In the 1990s and 2000s, many robotic systems were developed for robotic‐assisted surgery and are used in the clinic.^18^ With further progress in the last decade, the era of autonomous surgical robotics has begun.^12^

There is now a commercial version of a task‐autonomous robotic platform for cochlear implantation. The increasing degree of autonomy of such robotic platforms distinguishes them from already available robotic‐assisted surgery platforms. This increasing autonomy defines new categories of surgical instruments and raises important questions of patient acceptance, ethics and legality.^19^ Yang et al. recently proposed a classification system to categorize the level of autonomy of medical robotics, ranging from level 0 (no autonomy) to 5 (full automation of an entire surgery).^20^ While currently available robotic platforms represent level 0 to 1 (no autonomy to robotic assistance), a platform performing image‐guided cochlear access would be classified as level 2 (task‐autonomy).^20^

While numerous studies investigated the level of acceptance of robotic‐assisted surgery in surgeons,^14^, ^21^ data in patients are sparse and no study has characterized the acceptance of robotic surgery platforms above Yang level 1.

In this study, we aimed at evaluating the level of acceptance for task‐autonomous robotic surgery in a relevant patient population rather than a random patient sample. We therefore choose to survey patients and parents of infants scheduled for cochlear implantation. We found that the majority of patients and parents would consent to TARCI. Importantly, only a minority of patients would not want TARCI as their surgical procedure, even if their surgeon recommended it. Counterintuitively, those patients were significantly younger, which is interesting given the high incorporation of technology in the life of younger people. In contrast to this observation, a study by Boys et al. found no differences in the public perception of robotic‐assisted surgery between age groups.^22^ Another study by McDermott, however, found an overall fear and lack of trust in new technology in medicine in a young patient population with a mean age of 21.5 years.^23^ Notable limitations of those studies might be the participant selection based on convenience sampling and surveying without prior information on the topic. Noteworthy, surveyed parents, who were also significantly younger than patients, were highly accepting of TARCI. A possible explanation for this discrepancy could be the higher formal education of parents in our study. Education might enable a better understanding of the advantages of TARCI. Misunderstandings of possible advantages were identified when participants were asked for their level of acceptance in case of anomalous anatomy. While parents' level of acceptance increased, that of patients decreased, despite the benefit of precise planning of an image guided trajectory prior to the surgery increasing the safety of the procedure. Another interesting finding was that more parents thought family members would advise against TARCI and that this might influence their decision. However, if TARCI would be recommended by the surgeon for their child, all parents were likely to follow the recommendation. Regarding expectations towards TARCI, study subjects would expect a lower complication rate compared to manual cochlear implantation. Based on the limited number of TARCI procedures performed, no data on the complication rate compared to manual cochlear implantation are available to date. Notably, Alemzadeh et al. analysed adverse events in ∼1,7 million robotic‐assisted surgeries over a 14‐year period in the United States.^24^ They found adverse events related to the robotic system in 0.6% of cases, of which 76% were attributable to device malfunctions.^24^ For robotic platforms working at higher levels of autonomy, it will be exceedingly important to closely monitor and sufficiently report incidents. The expectations of shorter duration of the procedure contrasts reported procedure duration of ∼4 h (compared to 1.5 h for conventional cochlear implantation) in the first in man trial in nine patients.^25^ Of course, the duration of the procedure in this study was affect by the experimental setting of those first surgeries and includes intraoperative imaging and safety procedures. Undoubtedly, the procedure time will be significantly shortened, however, if it can be reduced by a factor of three to four remains to be seen.

Limitations of this study that need to be discussed are the small number of study subjects, particularly the number of parents, however, answers were homogenous. Second, patients were assumed to be unfamiliar with this procedure and thus reliant on education and decision guidance from their surgeon. Participant education of TARCI was standardized and designed to be unbiased. Emphasis was placed on the fact that the TARCI operates autonomously regarding the drilling process, with the surgeon monitoring the progress. Third, the evaluation of acceptance towards TARCI in this study was, by its nature, theoretical. However, in contrast to most studies on the acceptance of robotic surgery, we surveyed a defined patient population that is well informed on the topic and concerned with that surgery. Fourth, we did not asses if patients would be willing to pay an additional cost to undergo TARCI. As it is highly unusual to co‐pay for surgeries in Austria, we did not consider this question, however, it might be of high importance in other countries.

In conclusion, we found a high level of acceptance for TARCI, which was increased when TARCI was recommended by the surgeon. However, 16% of patients were unwilling to accept this technology after recommendation.

## CONFLICT OF INTEREST

All authors declare no conflict of interest.

## Supporting information

Supporting Material 1
