# Supplementary material for: Acceptance of patients towards task‐autonomous robotic cochlear implantation: An exploratory study
Source: Int J Med Robot. 2020 Oct 4;17(1):e2172. doi: 10.1002/rcs.2172 (PMC7900970; doi:10.1002/rcs.2172)
Supplement: Supplementary file 1 — Supporting Material 1 [file RCS-17-e2172-s001.docx]

**Fragebogen zur roboterchirurgischen Cochlea Implantation**

Bitte beantworten Sie die folgenden Fragen so ehrlich wie möglich. Ihre Antworten sind pseudonymisiert, das heißt, dass ihre Antworten nicht direkt mit ihrem Namen in Verbindung gebracht werden können.

**Hintergrund:** Es wird derzeit intensiv an der Entwicklung von Operationsrobotern für die Cochlea-Implantation gearbeitet, welche die Bohrung durch das Felsenbein (der Schädelknochen hinter der Ohrmuschel) zum Innenohr selbstständig durchführen. Erste Operationen an Patienten wurden im Rahmen einer klinischen Studie bereits erfolgreich durchgeführt. Der Vorteil dieser Methode liegt darin, dass bereits vor der Operation der Bohrkanal in einem dreidimensionalen Computertomogramm des Patienten geplant werden kann. Hierbei können wichtige Strukturen wie der Gesichts- und Geschmacksnerv, sowie das Innenohr bei der Operation geschont werden und es ist ein wesentlich kleinerer Bohrkanal nötig (Bild 1). Durch die Schonung des Innenohrs ist der Erhalt eines etwaigen Restgehörs am betroffenen Innenohr möglich. Der Roboter misst während der Bohrung ständig seine Position im Raum in Relation zum Schädelknochen, sowie Bohrwiderstand und Bohrtiefe. Ein Chirurg überwacht diesen Vorgang lediglich, kann die Bohrung gegebenenfalls aber jederzeit abbrechen. Eine direkte Steuerung während der Bohrung ist jedoch nicht möglich.

| Bild 1.: 3D – Rekonstruktion des Felsenbeins mit Planung des Bohrkanals |
| --- |
| 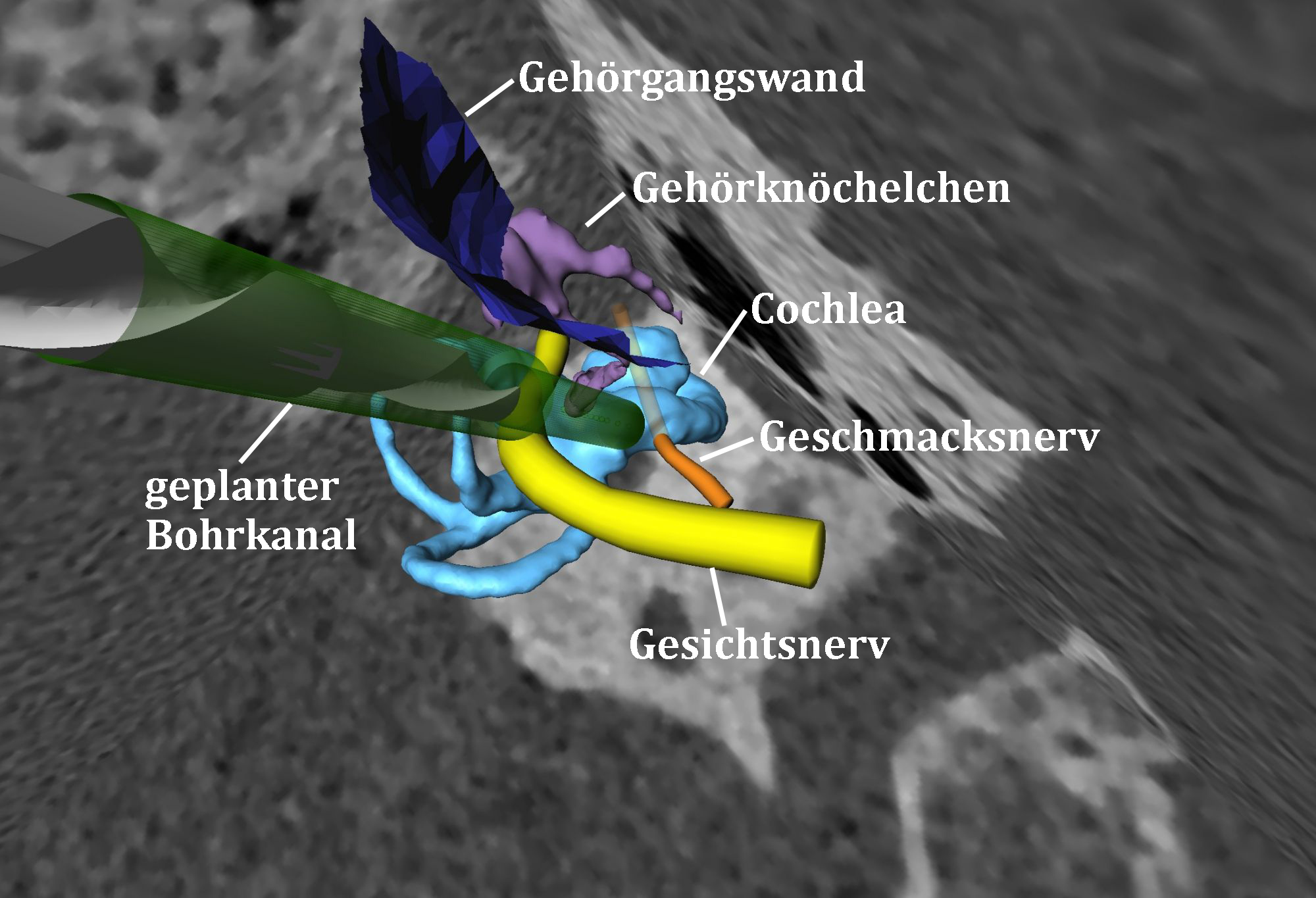 |
| Bild: Artorg Center for Biomedical Engineering Research, Universität Bern |

**- English translation for supplementary material only -**

**Questionnaire for robotic cochlear implantation**

Please answer the following questions as honestly as possible. Your answers are

pseudonymized, which means that your answers cannot be directly linked with your name.

**Background:** Scientists are currently working intensively on the development of surgical robots for cochlear implantation, which independently drill through the temporal bone (the skull bone behind the auricle) to the inner ear. The first operations on patients have already been successfully carried out as part of a clinical study. The advantage of this method is that the drill channel can be planned in a three-dimensional computed tomography of the patient before the operation takes place. Important structures such as the facial- and taste nerves as well as the inner ear can be spared during the operation and a much smaller drill channel is necessary (Fig. 1). By protecting the inner ear, it is possible to preserve residual hearing on the affected inner ear. During the drilling, the robot constantly measures its position in space in relation to the skull bone, as well as drilling resistance and drilling depth. A surgeon only monitors this process, but can interrupt the drilling at any time if necessary. However, direct control during drilling is not possible.

| Figure 1.: 3D – reconstruction of the temporal bone with planned drilling trajectory |
| --- |
| 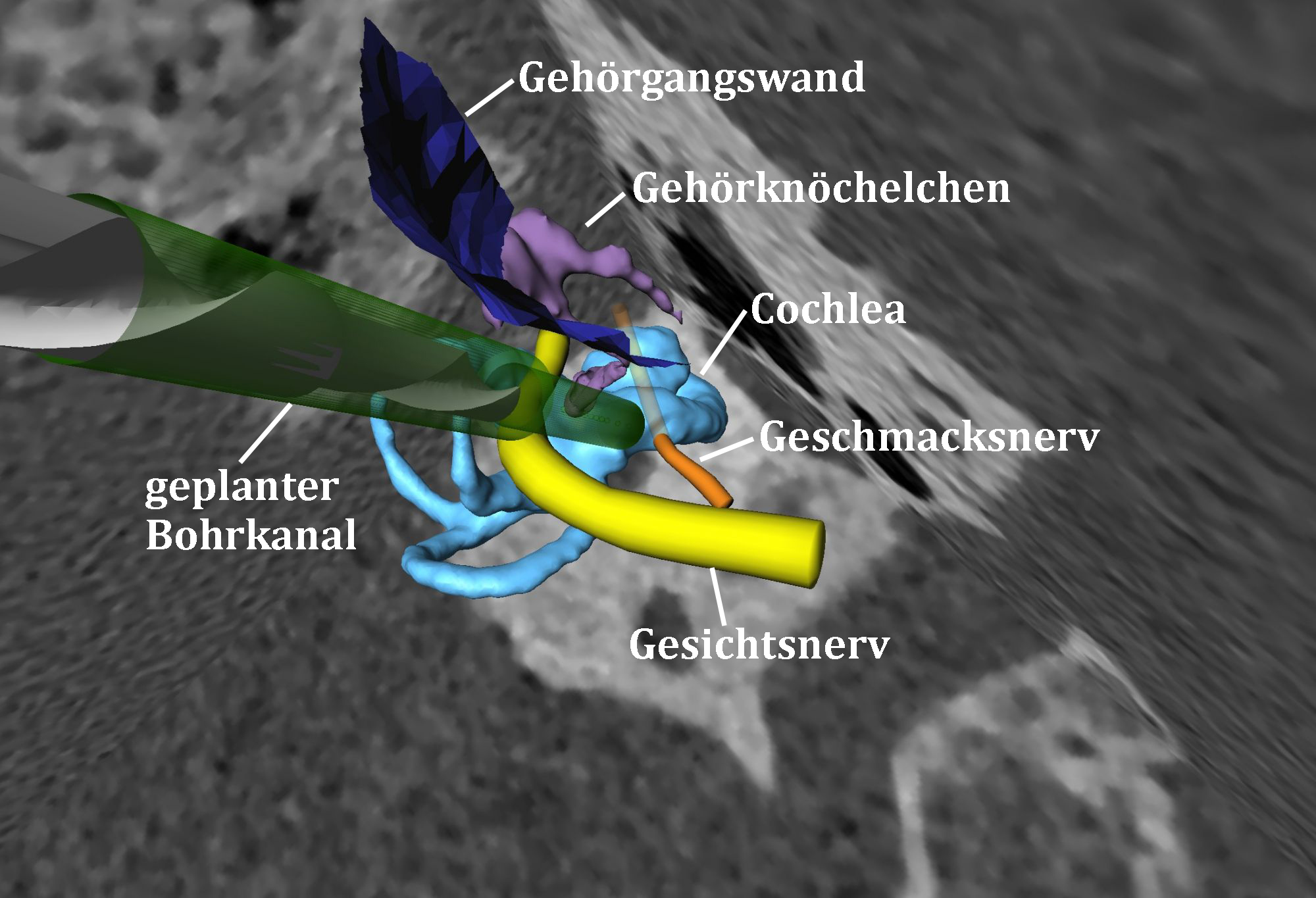 |
| Artorg Center for Biomedical Engineering Research, University Bern |
